# Fine Tuning of the Lactate and Diacetyl Production through Promoter Engineering in *Lactococcus lactis*

**DOI:** 10.1371/journal.pone.0036296

**Published:** 2012-04-27

**Authors:** Tingting Guo, Jian Kong, Li Zhang, Chenchen Zhang, Shumin Hu

**Affiliations:** State Key Laboratory of Microbial Technology, Shandong University, Jinan, People's Republic of China; Belgian Nuclear Research Centre SCK/CEN, Belgium

## Abstract

*Lactococcus lactis* is a well-studied bacterium widely used in dairy fermentation and capable of producing metabolites with organoleptic and nutritional characteristics. For fine tuning of the distribution of glycolytic flux at the pyruvate branch from lactate to diacetyl and balancing the production of the two metabolites under aerobic conditions, a constitutive promoter library was constructed by randomizing the promoter sequence of the H_2_O-forming NADH oxidase gene in *L. lacti*s. The library consisted of 30 promoters covering a wide range of activities from 7,000 to 380,000 relative fluorescence units using a green fluorescent protein as reporter. Eleven typical promoters of the library were selected for the constitutive expression of the H_2_O-forming NADH oxidase gene in *L. lactis*, and the NADH oxidase activity increased from 9.43 to 58.17-fold of the wild-type strain in small steps of activity change under aerobic conditions. Meanwhile, the lactate yield decreased from 21.15±0.08 mM to 9.94±0.07 mM, and the corresponding diacetyl production increased from 1.07±0.03 mM to 4.16±0.06 mM with the intracellular NADH/NAD^+^ ratios varying from 0.711±0.005 to 0.383±0.003. The results indicated that the reduced pyruvate to lactate flux was rerouted to the diacetyl with an almost linear flux variation via altered NADH/NAD^+^ ratios. Therefore, we provided a novel strategy to precisely control the pyruvate distribution for fine tuning of the lactate and diacetyl production through promoter engineering in *L. lactis*. Interestingly, the increased H_2_O-forming NADH oxidase activity led to 76.95% lower H_2_O_2_ concentration in the recombinant strain than that of the wild-type strain after 24 h of aerated cultivation. The viable cells were significantly elevated by four orders of magnitude within 28 days of storage at 4°C, suggesting that the increased enzyme activity could eliminate H_2_O_2_ accumulation and prolong cell survival.

## Introduction

*Lactococcus lactis* has long been used in dairy fermentation processes and considered as one of the most important starter cultures. It produces multifarious end metabolites during dairy fermentation, such as lactate, diacetyl, acetoin, vitamins and extracellular exopolysaccharides which contribute to the organoleptic and health-promoting properties of the fermented products [1]–[4]. *L. lactis* has become a model for rational industrial strain improvement because of its relatively small genome, simple metabolism and industrial relevance [5].

**Figure 1:**
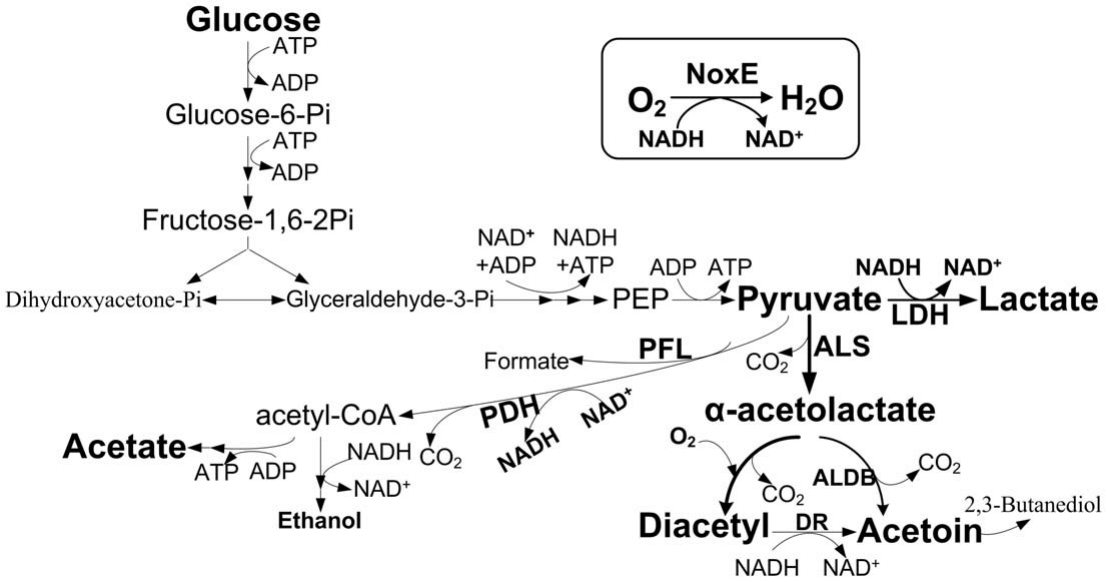
Metabolic pathway of glycolysis and diacetyl biosynthesis in L. lactis. PEP, phosphoenolpyruvate; LDH, lactate dehydrogenase; ALS, α-acetolactate synthase; ALDB, acetolactate decarboxylase; PDH, pyruvate dehydrogenase; DR, diacetyl reductase; PFL, pyruvate formate lyase; NoxE, H_2_O-forming NADH oxidase.

Diacetyl is an important aroma compound and essential for the flavor of dairy products. Normally, *L. lactis* undergoes homolactic fermentation, and most of the central intermediate pyruvate is converted to lactate, a reaction catalyzed by lactate dehydrogenase (LDH) with the oxidation of NADH to NAD^+^ for maintaining a redox balance [6]. Under aerobic conditions, the activities of α-acetolactate synthase (ALS) and NADH oxidase (NOX) are strongly increased [7]. ALS catalyzes the pyruvate to α-acetolactate. After decarboxylation, α-acetolactate is further converted to acetoin and diacetyl. The reoxidation of NADH by NOX would replace the role of the LDH in the regeneration of NAD^+^, allowing the accumulation of these two aroma compounds (Figure 1) [8]. However, in the presence of O_2_, *L. lactis* displays the metabolic shift from homolactic to mixed-acid product formation, including lactate, acetate and CO_2_. Hence, diacetyl accumulation is rather limited [9]. Therefore, several approaches to improve diacetyl production in *L. lactis* have been developed, such as the overexpression of the *als* and *nox-2* genes and the inactivation of the *ldh* and α-acetolactae decarboxylase (*aldB*) genes. The herein excessive pyruvate was channeled to acetoin or diacetyl via ALS, whereas the flux from pyruvate to lactate was almost abolished [8], [10]–[12]. Considering that lactate is an important metabolite that prevents fermented products from spoilage and contributes to the texture of dairy products, the strong overexpression or complete deletion of target genes limits the application of genetically modified lactic acid bacteria as starter cultures for industrial production of dairy products. Thus, strategies for the fine-tuning of gene expression are required to control the aimed metabolic fluxes.

Promoter engineering is interpreted as the creation of a functional promoter library for precisely controlling gene expression to perform metabolic optimization or control analysis. It has promising perspectives with respect to the research of functional genomics, cell network analysis and synthetic biology [13]–[15]. For example, a series of mutant *L. lactis* strains have been constructed based on synthetic promoters to demonstrate that LDH exerted virtually no control on the glycolytic flux at the wild-type enzyme level and the lactate production [16]. In addition, a synthetic promoter library has been used to evaluate the influence of different production levels of glucose-6-phosphate dehydrogenase on xylose fermentation and ethanol yield in *Saccharomyces cerevisiae* [17]. These previous studies have illustrated the valuable applications of the promoter engineering in the precise control and the quantitative assessment of gene expression level.

*L. lactis* is a facultatively anaerobic bacterium and O_2_ has negative effects on both cell growth and survival, because *in vivo* O_2_ is converted into reactive oxygen species (ROS) which cause protein, lipid and nucleic acid damage [18]. To cope with oxygen toxicity, *L. lactis* could grow via respiratory metabolism when heme is available [19]. Some lactic acid bacteria have antioxidant enzymes to detoxify O_2_-derived compounds [20]. Aside from the toxic effects of O_2_, aeration could induce the metabolic shift from homolactic to mixed-acid fermentation, making pyruvate metabolism more flexible. Therefore, lowering cytoplasmic O_2_ is economically significant in improving cell growth and survival under aerobic conditions.

Here we constructed a constitutive promoter library by randomizing the space sequence between the two conserved motifs of the promoter of the H_2_O-forming NADH oxidase (*noxE*) gene in *L. lactis*. Under the control of individual random promoters, the fine tuning of lactate and diacetyl production was achieved by precisely regulating the intracellular NADH/NAD^+^ ratios in *L. lactis*. Furthermore, the beneficial effects of the increased NoxE activity on cell survival were also investigated in this study.

## Materials and Methods

### Plasmids, bacterial strains and growth conditions

The plasmids used in this study are listed in Table 1. *E. coli* DH5α was grown aerobically in Luria Bertani broth at 37°C. The plasmid-free strain *L. lactis* MG1363 was used as host for the construction of the constitutive promoter library and considered as a wild-type strain in this study [21]. The *L. lactis* DA strain was a derivative of *L. lactis* MG1363 with the *aldB* gene deletion. *L. lactis* was grown in M17 broth (Oxoid, Basingstoke, United Kingdom) containing 0.25% (wt/vol) glucose (GM17) at 30°C. For milk fermentation, *L. lactis* was incubated in 12.5% (wt/vol) sterile, reconstituted skim milk (RSM) supplemented with 1% glucose (wt/vol). The following antibiotics were added at the indicated concentrations: chloramphenicol, 5 µg/mL for *L. lactis* or 10 µg/mL for *E. coli*; erythromycin, 10 µg/mL for *L. lactis*; and ampicillin, 100 µg/mL for *E. coli*.

**Table 1:** Plasmids used in this study.

Plasmid	Relevant characteristics	Reference or source
pMD18-T	Cloning vector, Amp ^R^	Takara
pT-GFP	*Bgl*II-*Pst*I-*gfp*-*Eco*RI fragment cloned in pMD18-T, Amp ^R^	This study
pSec∶Leiss∶Nuc	pWV01 replicon, expresses Nuc under PnisA control, Cm ^R^	[55]
pGFP	Promoter probing vector used in the construction of promoter library; pSec∶leiss∶Nuc derivative with PnisA and Nuc fragment replaced by *gfp* gene fragment, Cm ^R^	This study
pOgfp	pGFP derivative, carrying the native promoter O159, Cm ^R^	This study
pMgfp	pGFP derivatives, carrying individual random promoters of the promoter library, Cm ^R^	This study
pG^+^ host4	Derivative of pGK12 used for homologous recombination, Erm ^R^	[28]
pEnox	pMgfp derivative with *gfp* gene fragment replaced by *noxE* gene fragment, carrying eleven selected promoters, respectively, Cm ^R^	This study

### Construction of the constitutive promoter library

The primers used in this study are listed in Table 2. All molecular manipulations were performed as described previously [22]. *Taq* polymerase, restriction enzymes and T4 DNA ligase were used as stated by standard procedures (TaKaRa, Tokyo, Japan).

**Table 2:** Oligonucleotide primers used in this study.

Primer	Sequences (5′-3′)	Restriction sites
NOXEp-for	GGTAGATCTTTTGATTCAGAAACTATGTGG	*Bgl*II
NOXEp-rev	GATCTGCAGACTAATAGGTCTCCTTTA	*Pst*I
NOXEp-mut	CGGAGATCTNNNNNNNNTTGACANNNNNNNNNNNNNNNNNNNTANAATNNNNNTTTCACAATGTTCACAAGCGCTTAC	*Bgl*II
GFP-for	TTCTGTCAGTGGAGAGGGT	
GFP-rev	GGATAACGGGAAAAGCATT	
GAP-for	GCGACAGGTTTCTTTGCGA	
GAP-rev	CGTCTGCCATTGGTGCTAA	
NOXE-for	CCTCTGCAGGTATGAAAATCGTAGTTATC	*Pst*I
NOXE-rev	TTCGTCGACTTATTTGGCATTCAAAGCT	*Sal*I
TU-for	ACTCTCGAGCACTAAAATGCGTCAGTCAAT	*Xho*I
TU-rev	GGCGAATTCATTTCTCTTTTCTATCTCAT	*Eco*RI
TD-for	GCGGAATTCGATATTGATGTAGCTGA	*Eco*RI
TD-rev	TTGCGGCCGCTCCACTATCTATAAAATG	*Not*I
DC-for	ATAATGAATCAGTCGAATGCAAGA	
DC-rev	TTTGGGCAATCCAGCAACTCCTA	

The restriction sites in the primer sequences are underlined.

**Figure 2:**
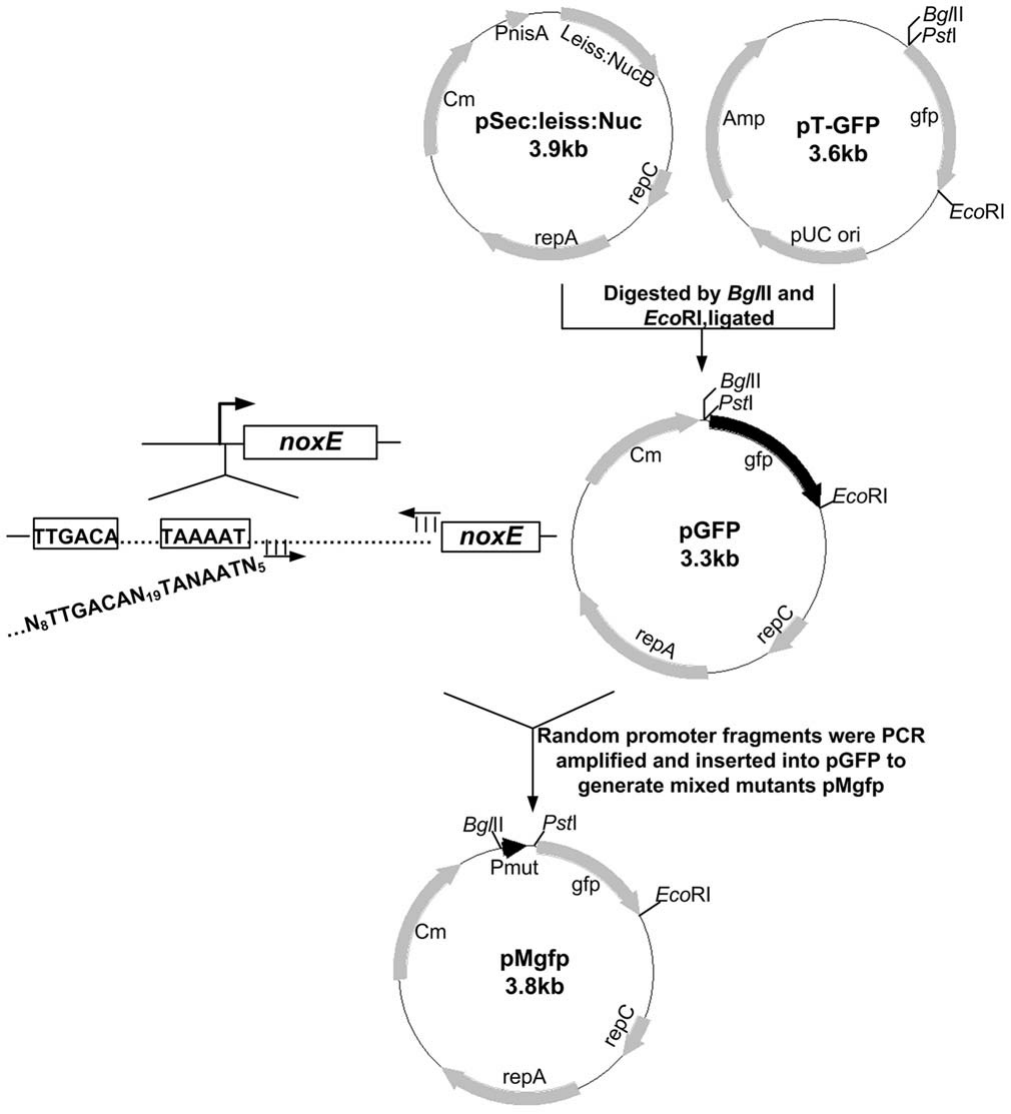
Scheme for generating the constitutive promoter library in *L. lactis*. N = A, G, T or C.

The scheme for generating the promoter library is shown in Figure 2. The nisin-inducible promoter PnisA and *nuc* gene fragment of the *E. coli*/*L. lactis* shuttle vector pSec∶Leiss∶Nuc was replaced by the promoterless green fluorescent protein (*gfp*) gene fragment, resulting in the promoter probing vector pGFP.

The *noxE* promoter fragment was PCR amplified from the genomic DNA of *L. lactis* MG1363 with primers NOXEp-for and NOXEp-rev. The PCR product was digested with *Bgl*II and *Pst*I and ligated to compatible ends of the digested pGFP to generate pOgfp. To develop the constitutive promoter library for *L. lactis*, the fragments containing randomized promoters were amplified using primers NOXEp-mut and NOXEp-rev by degenerate PCR with the plasmid pOgfp DNA as template. After digested with *Bgl*II and *Pst*I, the random promoter fragments were inserted into the corresponding sites of the probing vector pGFP to generate the mixed plasmids pMgfp. The mixed plasmids pMgfp were then electrotransformed into the competent cells of *L. lactis* MG1363, and the cells were plated onto the GM17 agar containing chloramphenicol [23]. The colonies ranging in color from white to dark green were picked from the GM17 agar plates. After overnight static incubation at 30°C, each culture was diluted 100-fold in 5 mL of fresh GM17 medium and further incubated for 10 h at 30°C. The culture turbidity was monitored by OD600. Cells were collected from 1 mL cultures by centrifugation at 7,000 g for 5 min, washed twice and resuspended in 500 µL of phosphate buffered saline (PBS, 137 mM NaCl, 2.7 mM KCl, 10 mM Na_2_HPO_4_, and 2 mM KH_2_PO_4_, pH 7.4). The fluorescence intensity of the suspension was determined by an LS-50B spectrofluorometer (PerkinElmer) with excitation at 488 nm and emission at 511 nm. To obtain a relative fluorescence unit (RFU), the fluorescence intensity of the cells carrying the promoter probing vector pGFP was used as a background and subtracted from that of cells containing plasmids pMgfp.

### Characterization of the constitutive promoter library

Eleven colonies with various promoter activities were selected and their promoters were sequenced. The promoter strength was evaluated as described by Alper *et al.* [13]. Growth was monitored by OD600 every thirty minutes over five hours. At the same intervals, the cultures were sampled and the fluorescence intensities were measured as above. The slope of the fluorescence versus culture turbidity was considered as the exponential growth phase steady-state concentration of GFP.

The cultures in GM17 medium were collected at the exponential phase and the total RNA was extracted with a RNA simple Total RNA Kit (TIANGEN, Beijing, China) according to the manufacturer's protocols. The quantity and purity of RNA were determined spectrophotometrically at 260 nm and 280 nm. Reverse transcription was performed with Random 6 mers and Oligo dT primer using the PrimeScript RT reagent Kit (TaKaRa, Tokyo, Japan) according to the manufacturer's instructions. Real-time PCR was performed with the SYBR Premix Ex TaqII (TaKaRa, Tokyo, Japan) applying the protocol in the Real-Time PCR Detection Systems (Bio-Rad, Hercules, CA, USA). The *gfp* transcript was PCR amplified with the primers GFP-for and GFP-rev. The *gapA* (D-glyceraldehyde-3-phosphate dehydrogenase) transcript used as internal standard was amplified with the primers GAP-for and GAP-rev.

### Expression of the noxE gene with selected promoters

The *noxE* gene was PCR amplified from the genomic DNA of *L. lactis* MG1363 using primers NOXE-for and NOXE-rev. The PCR product was digested by *Pst*I and *Sal*I and inserted into the same sites of vector pMgfp to replace the *gfp* gene, yielding pEnox (E: eleven individual promoters selected). Subsequently, the plasmids were introduced into *L. lactis* DA to obtain recombinant *L. lactis* DA/pEnox, respectively. The expression of the *noxE* gene under the control of eleven promoters was analyzed via the intracellular NoxE activity assay. See below for details.

### Fermentation conditions and analytical methods

The recombinant strains were pre-cultured in 5 mL of GM17 medium, and 2 mL of the overnight cultures were incubated in 500-mL Erlenmeyer flasks with 100 mL of GM17 medium with 200 rpm orbital shaking at 30°C.

Cell growth was monitored by OD600. Glucose, acetate, lactate, formate and ethanol were analyzed by high-performance liquid chromatography (HPLC; Shimazu, Japan) using a column of Aminex HPX-87H Ion Exclusion particles (300 mm×7.8 mm, Bio-Rad, Hercules, CA, USA), at a column temperature of 65°C with 5 mM sulfuric acid as the mobile phase at a flow rate of 0.6 mL/min. Acetoin, α-acetolactate and diacetyl were determined according to Benson et al. [24]. H_2_O_2_ was measured using the H_2_O_2_ quantified analysis Kit (Sangon Biotech, Shanghai, China). NADH and NAD^+^ were extracted as described previously [25], and their concentrations were measured by enzyme cycling assay [26], [27].

### Enzymatic measurements

Cells from 5 mL cultures were collected by centrifugation and washed twice with potassium phosphate buffer (pH 7.0). The cells were resuspended in 2.5 mL of potassium phosphate buffer and disrupted by sonication on ice (400 w, sonication for 3 s, intermission for 8 s). The supernatant was recovered by centrifugation at 12,000 g for 10 min to determine the *in vivo* enzyme activities. The protein concentration was determined using the Bradford protein assay with bovine serum albumin as standard.

The NoxE activity was determined spectrophotometrically by measuring the initial rate of NADH oxidation at 25°C. The total 200 µL assay mixture contained 0.3 mM NADH and 0.3 mM EDTA in 50 mM potassium phosphate buffer. The reaction was initiated by adding 10 µL of cell extract and monitored by the decrease at OD340nm using Spectra MAX 190 (Molecular Devices Corporation, U.S.A.). A unit enzyme was defined as the amount which catalyzed the oxidation of 1 µmol of NADH to NAD^+^ per minute at 25°C. LDH activity was determined according to the method of Andersen et al. [16].

### Deletion of the aldB gene

The upstream and downstream sequences of the *aldB* gene were PCR amplified from the genomic DNA of *L. lactis* MG1363 with primers TU-for/TU-rev and TD-for/TD-rev, and then inserted into the vector pG^+^host4, respectively. Subsequently, the resulting plasmid was used to perform homologous double-cross in the *L. lactis* MG1363 chromosome as the modified method described previously [28]. Gene deletion was verified by PCR amplification with the primer DC-for and DC-rev.

## Results

### Construction of the constitutive promoter library

The sequence alignment of the *noxE* promoter located at *L. lactis* MG1363 genome with the consensus promoter sequences of *L. lactis* revealed that the −35 region had high identity with the TTGACA sequence, whereas the −10 region was less conserved with the sequence TAAAAT deviating from the canonical TATAAT sequence in the 3rd position. In our strategy, the −35 region nucleotides were kept constant and the −10 region was generally maintained except the 3rd base A was randomized, yielding TANAAT (N = A, T, G or C). The spacing between the −10 and −35 region had 19 completely random nucleotides. Furthermore, the randomization was also introduced into 8 bases upstream of the −35 region and 5 bases downstream of the −10 region for higher diversity of random promoters.

**Figure 3:**
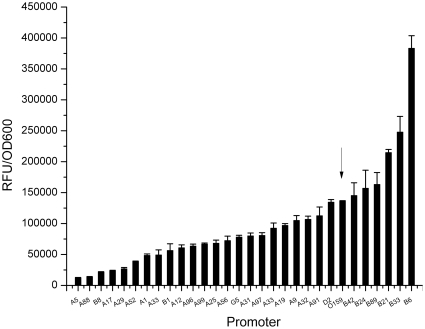
Promoter library for constitutive gene expression in *L. lactis*. The activity of the promoter is measured as RFU per OD600. The data are the means and standard deviations of results from five independent experiments. The arrowhead indicates the native promoter O159.

To screen the random promoters easily, a promoter probing vector pGFP was constructed using GFP as reporter based on the *E. coli/L. lactis* shuttle vector pSec∶leiss∶Nuc. The randomized promoter fragments were inserted into pGFP, and the resulting plasmids pMgfp were transformed into *L. lactis* MG1363. Five hundred colonies of *L. lactis* MG1363/pMgfp carrying individual random promoters were picked by observation of the green color on the GM17 plates. To assess the promoter activities, the fluorescence intensities of the colonies were determined and 30 representative random promoters were selected to form a constitutive promoter library (Figure 3). The promoter activities of the library spanned from 7,000 to 380,000 RFU, covering 3 to 4 logs of expression levels in small increments. Six random promoters showing higher activity than the native promoter O159 were obtained, and the most potent promoter B6 exhibited a 2.8-fold activity increment.

### Characterization of the promoter library

**Figure 4:**
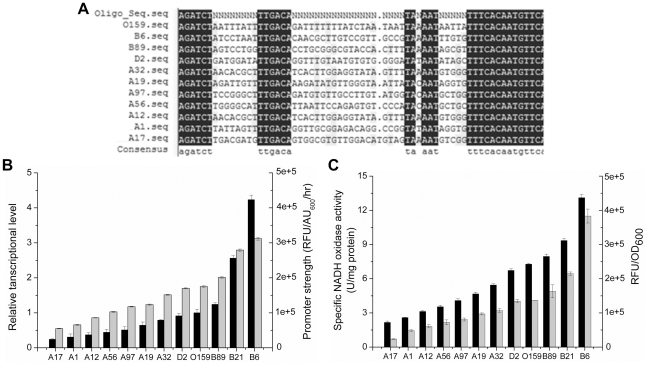
Characterization of the promoter library. (A) Sequence analysis of the selected promoters. N = A, G, T or C. (B) Determination of promoter strength from the transcript quantification of the *gfp* gene (black) and dynamics of GFP production based on fluorescence intensity (gray). (C) Specific NoxE activity (black) under the control of eleven selected promoters (promoter activity is shown as gray). In panels (B) and (C) the values are means ± standard deviations of three independent experiments.

Eleven promoters (B6, B21, B89, D2, A32, A19, A97, A56, A12, A1 and A17) with activities covering from 23,000 to 380,000 RFU were selected for the sequence analysis. As shown in Figure 4A, all selected promoters had a DNA sequence identical to specific sequence of the designed oligonucleotide NOXEp-mut. No base change was observed in the −35 region, while base changes were found in the −10 region of five promoters which exhibited lower activity than that of the native promoter O159. The base T insertion was observed in the spacer of promoter A17, resulting in a drastic reduction in promoter activity. The alignment showed that the higher similarity of sequences outside the conserved regions between the random promoter and the native led to stronger promoter activity.

To eliminate the effects of protein maturation, degradation and cell growth rate on the GFP fluorescence intensity, the eleven promoters strength were determined by a dynamic model in which the influencing factors were comprehensively considered [13]. Based on this model, the promoter strength of the library varied from 55,006±79 to 311,982±410 and increased by 17.51% on average between the adjacent promoters (Figure 4B).

To characterize the promoter library at transcriptional level, relative mRNA levels of the *gfp* transcripts under the control of eleven promoters were analyzed by Real-Time PCR. The relative level of the *gfp* transcript spanned a 17.49-fold variation and correlated well with the promoter strength (Figure 4B).

### Tuning of the lactate and diacetyl production in L. lactis

To investigate the regulation capacity of different promoters on the distribution of the pyruvate flux to lactate and diacetyl, the sequenced promoters were fused with the *noxE* gene from *L. lactis* MG1363. Then, the resulting plasmids (pB6nox, pB21nox, pB89nox, pD2nox, pA32nox, pA19nox, pA97nox, pA56nox, pA12nox, pA1nox and pA17nox) were introduced into *L. lactis* DA which was unable to convert α-aectolactate to acetoin by acetolactate decarboxylase. The NoxE activities of the recombinant strains were determined at the late exponential phase. As expected, the NoxE activity of *L. lactis* DA was very low (0.23±0.05 U/mg protein). In the eleven recombinants, it showed linear increase with the promoter strength, from 2.17±0.12 U/mg protein to 13.11±0.28 U/mg protein (Figure 4C). However, the differences in the enzyme activity between the adjacent promoters did not exceed 1.5 units except promoter B6 which was 3.8 units higher than the next promoter B21. Moreover, the LDH activity of the recombinant strains was very similar to that of wild-type (7.21±0.14 U/mg protein).

After 12 h of aerobic culture in GM17 medium, the cell growth and glucose consumption rate of *L. lactis* DA and eleven recombinant strains were similar to those of the wild-type *L. lactis* MG1363, indicating that genetic modification had little influence on *L. lactis* growth. As shown in Table 3, the intracellular NADH/NAD^+^ ratios varied from 0.711±0.005 to 0.383±0.003 by controlling the expression of the NoxE. The lactate accumulation exhibited a nearly linear decrease from 21.15±0.08 mM to 9.94±0.07 mM, whereas the diacetyl yield showed a gradual increase from 1.07±0.03 mM to 4.16±0.06 mM. These results indicated that the decreased pyruvate flux to the LDH pathway was rerouted to the ALS pathway accompanied by the enhancement of NoxE activity. Meanwhile, acetate yield in the recombinant strains was on average 2.48 mM higher than that of the strain *L. lactis* DA and acetoin accumulation was still detectable in the recombinant strains. In the *aldB*-deficient strains, the unstable intermediate α-acetolactate was directly converted to diacetyl, which was subsequently reduced to acetoin by diacetyl reductase in the presence of O_2_. The acetoin and diacetyl yields were undetectable in *L. lactis* DA, whereas the lactate and acetate production were 23.41±0.11 mM and 8.47±0.47 mM, respectively.

**Table 3:** pH, product and NADH/NAD^+^ ratios of different recombinant strains at initial growth pH of 7.3 after 12 h aerobic culture.

		Concentrations of pyruvate metabolites (mM)				
Strains	Final pH	Lactate	Acetate	Diacetyl	Acetoin	NADH/NAD^+^ ratio
DA	5.93±0.03	23.41±0.11	8.47±0.14	ND	ND	0.741±0.009
DA/pA17nox	6.02±0.04	21.15±0.08	8.40±0.15	1.07±0.03	0.69±0.02	0.711±0.005
DA/pA1nox	6.05±0.02	20.28±0.13	8.43±0.09	1.19±0.01	0.87±0.05	0.689±0.006
DA/pA12nox	6.05±0.05	19.86±0.29	9.26±0.1	1.51±0.03	0.92±0.03	0.681±0.002
DA/pA56nox	6.07±0.02	19.09±0.12	9.12±0.14	1.8±0.01	1.01±0.04	0.556±0.008
DA/pA97nox	6.10±0.01	18.15±0.21	10.29±0.13	1.8±0.04	1.05±0.01	0.55±0.005
DA/pA19nox	6.10±0.01	17.46±0.17	11.08±0.07	1.84±0.05	1.05±0.03	0.541±0.005
DA/pA32nox	6.12±0.02	16.24±0.1	11.96±0.08	2.02±0.04	1.38±0.01	0.462±0.002
DA/pD2nox	6.14±0.04	14.21±0.13	13.11±0.12	2.26±0.02	1.67±0.05	0.457±0.002
DA/pB89nox	6.18±0.01	13.00±0.09	13.42±0.21	2.39±0.06	1.96±0.04	0.413±0.004
DA/pB21nox	6.22±0.02	11.76±0.16	13.09±0.01	3.38±0.12	2.14±0.04	0.406±0.003
DA/pB6nox	6.32±0.03	9.94±0.07	12.34±0.14	4.16±0.06	2.83±0.06	0.383±0.003

ND, not detected. The values are means ± standard deviations for three independent experiments.

**Figure 5:**
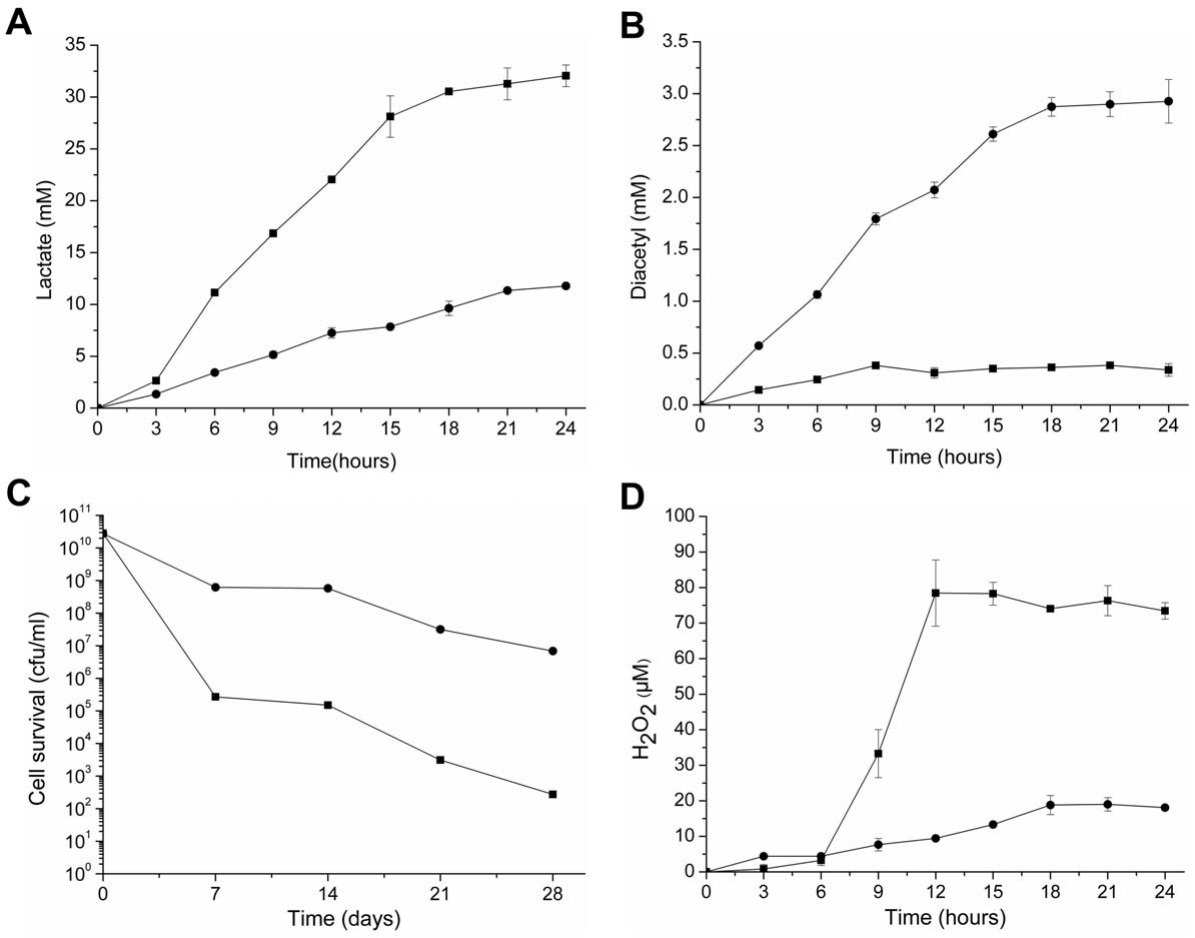
Metabolite accumulation and cell survival of *L. lactis* DA and the recombinant strain. Lactate production (A) and diacetyl production (B) by strains *L. lactis* DA (▪) and *L. lactis* DA/pB6nox (•) in RSM added 1% (wt/vol) glucose. Cell survival (C) and H_2_O_2_ accumulation (D) in *L. lactis* DA (▪) and *L. lactis* DA/pB6nox (•) after aerobic cultivation in GM 17 medium. In panels (A), (B), (C) and (D) the values are means ± standard deviations of three independent experiments.

Among the eleven recombinant strains, *L. lactis* DA/pB6nox consumed 33.96% and 23.88% of the carbon flux towards lactate and diacetyl under aerobic conditions, respectively. After *L. lactis* DA/pB6nox incubation in RSM supplemented with 1% glucose for 24 h, lactate production was 11.78±0.32 mM and diacetyl production was 2.93±0.21 mM with the pH decreasing from 6.63 to 5.02±0.01. The lactate yield in *L. lactis* DA was 32.05±1.05 mM with the pH decreasing from 6.61 to 4.67±0.03, whereas little diacetyl was accumulated (Figure 5A and 5B).

### Cell survival and H_2_O_2_ accumulation

To evaluate the effects of increased NoxE activity on the viability of *L. lactis* under aerobic conditions, *L. lactis* DA and *L. lactis* DA/pB6nox were cultured aerobically for 24 h, after which the cultures were transferred to 4°C and cell viability was examined at intervals over one month. As shown in Figure 5C, initially, the cell populations of the two cultures reached approximately 2.0×10^10^ per mL. Viable counts of *L. lactis* DA dynamically dropped as time progressed, and a 10^8^-fold decrease was observed after 28 days of storage. Comparatively, the viability of the *L. lacits* DA/pB6nox culture remained approximately 6.8×10^6^ cells per mL, four orders of magnitude higher than that of *L. lactis* DA.

The H_2_O_2_ concentration of the *L. lactis* DA and *L. lactis* DA/pB6nox cultures were tested to elucidate the cause of cell death under aerobic conditions. As shown in Figure 5D, in *L. lactis* DA, H_2_O_2_ accumulated rapidly within 6 h of incubation and reached a concentration of 78.44±9.29 µM after 24 h. In contrast, H_2_O_2_ accumulation was below 10 µM during 12 h of cultivation in *L. lactis* DA/pB6nox. After 24 h, the final H_2_O_2_ concentration of *L. lactis* DA/pB6nox was 18.08±0.33 µM, which was 76.95% lower than that of *L. lactis* DA. The reduction in the H_2_O_2_ concentration of *L. lactis* DA/pB6nox might have resulted from the elevated NoxE activity which catalyzed the oxidation of NADH by simultaneously reducing O_2_ to H_2_O.

## Discussion

With the advent of metabolic engineering, *L. lactis* has received increasing attention with the aim to promote the flavor and health advantages of fermented products, through the production of, for example, homoalanine, diacetyl, mannitol and folate [10], [29]–[33]. This study provided a platform for precisely regulating the metabolic flux via promoter engineering instead of the gene inactivation or overexpression in *L. lactis*. The partial redistribution of the pyruvate flux from lactate to diacetyl was achieved by controlling the *noxE* gene expression through a constitutive promoter library in *L. lactis*. Furthermore, we newly demonstrated that the elevated NoxE activity had a positive role in eliminating H_2_O_2_ and prolonging the cell-survival of *L. lactis*.

Sequence alignment showed that the promoter of the *noxE* gene from *L. lactis* MG1363 possessed the typical promoter properties [34]. Therefore, the mutant strategy was performed to randomize the space sequence of the *noxE* promoter based on the previous method [35]. A total of 30 random promoters from 500 mutants were selected to form the constitutive promoter library, which displayed broad variability with small steps of activity change between 0.1 and 2.8-fold of the native promoter. Sequence analysis verified that any alteration of the bases in the conserved motifs and changes in the spacer length could lead to a drastic decrease of promoter strength, which supported the postulates in the previous report [36]. Moreover, the sequences outside the −10 and −35 region may influence promoter strength. Eleven typical promoters were used to confirm the effective and stable characteristics of the library through promoter strength measurements and the mRNA transcript levels of the *gfp* gene. In addition, the *noxE* gene was used to prove the broad application range of the promoter library. NoxE activity showed a nearly linear correlation with promoter activity. Consequently, we developed a constitutive promoter library with a wide promoter activity range for fine-tuning of gene expression in *L. lactis*, regardless of the target gene context.

Although the nisin controlled gene expression (NICE) system has been utilized extensively in *L. lactis* [37], some shortcomings confined it to laboratory experimental conditions, including inducer usage, expression delay and heterogeneity of transcription levels in cell population [35]. Therefore, practical and stable properties of constitutive gene expression systems are desired in large-scale processes. The stable expression of GFP and NoxE driven by random promoters confirmed that the constitutive promoter library could meet the demands of the industrial fermentation process.

Cofactors are essential in completing a large number of biochemical reactions, and their manipulation has been proved to have great influences on metabolic networks [38]. The H_2_O-forming NADH oxidase specifically utilizes NADH and provides an extra route for the regeneration of NAD^+^ when O_2_ is available [39], [40]. In this study, eleven typical promoters from the promoter library were chosen to precisely control the *noxE* expression and the intracellular NADH/NAD^+^ ratios were pinpointly regulated. The direct oxidation of NADH necessary for pyruvate reduction by the increased NoxE activity resulted in a diminished pyruvate flux towards lactate via LDH, and the pyruvate flux was redistributed to the ALS pathway. Subsequently, α-acetolactate was decarboxylated into diacetyl in the presence of O_2_. The Metabolic Control Analysis (MCA) prediction and experimental observation showed that the glycolytic flux to the α-acetolactate branch was less than 0.1% in wild-type *L. lactis* [41]. However, the increasing NoxE activity driven by the eleven promoters led to the increase of 5.98% to 23.88% flux towards α-acetolactate and retained 67.29% to 33.96% flux to lactate. Acetate production exhibited a slight increase, probably due to the specific PDH activity which catalyzed the conversion of pyruvate to acetyl-CoA with the regeneration of NADH under aerobic conditions [42]. Moreover, neither formate nor ethanol was detected, indicating that no flux was distributed to the pyruvate formate lyase pathway and the alcohol dehydrogenase pathway, which was in agreement with the previous report [43]. In the milk fermentation process, the carbon flux was apportioned to lactate and diacetyl in 4∶1 proportion in *L. lactis* DA/pB6nox, in which the diacetyl yield was significantly improved as compared to the wild-type strain. Accordingly, through the precise control of the *noxE* gene expression levels by the constitutive promoter library, the tight constraint on the end-product fluxes in the wild type was alleviated by the gradual lowering of NADH/NAD^+^ ratios, yielding a series of recombinant strains with small differences in the proportion of lactate and diacetyl production among the end metabolites, which provide potential strains to optimize metabolite distribution.

Generally, the tolerance of lactic acid bacteria to O_2_ requires the presence of either catalase, NADH oxidases (dehydrogenase), superoxidase dismutase (SOD) or thiol-active enzyme system [44]. There are more than seven NADH oxidase and dehydrogenase genes in the *L. lactis* genome, including *noxA*, *noxB*, *noxC*, *noxD*, *noxE*, *yphA* and *aphF* [45], [46]. NoxA and NoxB are two membrane-integrated NADH-dehydrogenases and have been demonstrated to be components of the electron transfer chain (ETC) [47]. NoxC and NoxD are described as H_2_O_2_-forming NADH oxidases, however there is no experimental evidence to support this [48]. NoxE is a well characterized H_2_O-forming NADH oxidase [39]. The *yphA* gene encodes a NADH dehydrogenase similar to that of *Aquifex aeolicus*, which participates in aerobic energy metabolism [45]. AhpF is an H_2_O_2_-forming NADH oxidase, constituting the alkyl hydroperoxide reductase (AhpR) system [49]. Whole-genome transcriptome analysis has revealed that the H_2_O_2_-forming NADH oxidase and SOD were induced to alleviate oxidative stress under aerobic conditions, resulting in H_2_O_2_ accumulation [50]. In addition, a small amount of H_2_O_2_ in aerated cultures of *L. lactis* may also result from pyruvate oxidase activity (POX, encoded by the *poxL* gene) [51]. However, *L. lactis* is catalase-negative, so the considerable H_2_O_2_ accumulation leads to oxidative damage [52]. Figure 5C and 5D showed that the H_2_O_2_ concentration of the recombinant strain *L. lactis*DA/pB6nox was markedly reduced and the cell viability was significantly elevated compared with the wild-type strain. This variance could be the result of the elevated NoxE activity in the recombinant strain. Firstly, more dissolved O_2_ was consumed in the H_2_O-forming reaction by the elevated NoxE activity, which reduced the substrate O_2_ involved in the H_2_O_2_-forming reaction. Secondly, in *L. lactis*, NADH can be consumed by NoxE, LDH and H_2_O_2_-forming NADH oxidase. Because NoxE possesses much higher affinity for NADH (*Km* = 4.1 µM) than the other H_2_O_2_-forming NADH oxidase (for example, the *Km* value of NADH for AhpF was 76 µM), it succeeded in competing with the H_2_O_2_-forming NADH oxidase for the substrate NADH, leading to lower H_2_O_2_ production [39], [53]. Moreover, although the *Km* value of the LDH (10 µM) for NADH could compare with that of the NoxE, there is no H_2_O_2_ generation in the reaction catalyzed by LDH. Subsequently, decreased H_2_O_2_ accumulation could effectively prevent the formation of HO^·^ via the Fenton reaction and reduce ROS [54], therefore enabling *L. lactis* to be resistant to oxidative stress for achieving long-term cell survival.

In conclusion, here promoter engineering was successfully used to avoid the disadvantages brought by the massive expression of controlling enzyme and elimination of the branching flux. This study proved that promoter engineering was a useful genetic toolbox for metabolic pathway analysis and optimal metabolite distribution. As rapid acidification and flavor compound generation are crucial criteria for starter cultures of lactic acid bacteria, the recombinant strains constructed in this study showed both reasonable ratios of end products and long-term cell survival, which opens perspectives for rational improvement of starter cultures in dairy fermentation industry.
